# Activation of the endoplasmic reticulum stress response in skeletal muscle of G93A*SOD1 amyotrophic lateral sclerosis mice

**DOI:** 10.3389/fncel.2015.00170

**Published:** 2015-05-18

**Authors:** Dapeng Chen, Yan Wang, Eva R. Chin

**Affiliations:** ^1^School of Public Health, University of MarylandMD, USA; ^2^Proteomics Core Facility, College of Computer, Mathematics and Natural Sciences, University of MarylandMD, USA

**Keywords:** amyotrophic lateral sclerosis, skeletal muscle, endoplasmic reticulum stress, misfolded proteins, muscle atrophy, protein synthesis, unfolded protein response

## Abstract

Mutations in Cu/Zn superoxide dismutase (SOD1) are one of the genetic causes of Amyotrophic Lateral Sclerosis (ALS). Although the primary symptom of ALS is muscle weakness, the link between SOD1 mutations, cellular dysfunction and muscle atrophy and weakness is not well understood. The purpose of this study was to characterize cellular markers of ER stress in skeletal muscle across the lifespan of G93A*SOD1 (ALS-Tg) mice. Muscles were obtained from ALS-Tg and age-matched wild type (WT) mice at 70d (pre-symptomatic), 90d and 120–140d (symptomatic) and analyzed for ER stress markers. In white gastrocnemius (WG) muscle, ER stress sensors PERK and IRE1α were upregulated ~2-fold at 70d and remained (PERK) or increased further (IRE1α) at 120–140d. Phospho-eIF2α, a downstream target of PERK and an inhibitor of protein translation, was increased by 70d and increased further to 12.9-fold at 120–140d. IRE1α upregulation leads to increased splicing of X-box binding protein 1 (XBP-1) to the XBP-1s isoform. XBP-1s transcript was increased at 90d and 120–140d indicating activation of IRE1α signaling. The ER chaperone/heat shock protein Grp78/BiP was upregulated 2-fold at 70d and 90d and increased to 6.1-fold by 120–140d. The ER-stress-specific apoptotic signaling protein CHOP was upregulated 2-fold at 70d and 90d and increased to 13.3-fold at 120–140d indicating progressive activation of an apoptotic signal in muscle. There was a greater increase in Grp78/BiP and CHOP in WG vs. the more oxidative red gastrocnemius (RG) ALS-Tg at 120–140d indicating greater ER stress and apoptosis in fast glycolytic muscle. These data show that the ER stress response is activated in skeletal muscle of ALS-Tg mice by an early pre-symptomatic age and increases with disease progression. These data suggest a mechanism by which myocellular ER stress leads to reduced protein translation and contributes to muscle atrophy and weakness in ALS.

## Background

Amyotrophic Lateral Sclerosis (ALS) is a fatal motor neuron disease characterized by degeneration of motor neurons and progressive paralysis of skeletal muscle. ALS is inevitably fatal, with patients generally dying due to respiratory failure within 2–5 years of diagnosis (Rowland and Shneider, 2001). Although the majority of ALS cases are sporadic without family history, 5–10% of the total cases of ALS have a known genetic basis (Rowland and Shneider, 2001). Mutations in human Cu/Zn superoxide dismutase 1 (SOD1) account for ~20% of familial ALS (fALS) cases (Rosen et al., 1993). Mice generated to express a human Cu/Zn SOD1 mutation found in fALS patients (Gly^93^ to Ala; G93A) develop a rapidly progressive and fatal motor neuron disease similar to the clinical phenotype of ALS (Gurney et al., 1994). There is evidence that the SOD1 mutations exert their deleterious effects through a “gain-of-function” mechanism rather than through a loss of superoxide dismutase activity (Yim et al., 1996). The nature of this toxic “gain-of-function” is not known, although a number of putative mechanisms have been proposed, including oxidative stress, glutamate-mediated excitotoxicity, mitochondrial dysfunction, protein aggregation and endoplasmic reticulum (ER) stress (Robberecht and Philips, 2013).

Neurodegenerative diseases, including ALS, that result from unfolded/misfolded proteins have been linked to ER stress (Kaufman, 1999). Most newly synthesized proteins are folded properly in the ER, but unfolded and misfolded proteins accumulate in the ER lumen, causing cellular stress, activation of unfolded protein response (UPR) and an ER stress response (Xu et al., 2005). The ER stress response involves activation of three ER-resident stress sensors: protein kinase RNA-activated-like ER kinase (PERK), inositol-requiring kinase 1-alpha (IRE1α), and activating transcription factor 6 (ATF6; Xu et al., 2005). Normally, these ER stress sensors physically interact with the ER chaperone immunoglobulin binding protein (Grp78/BiP) which suppresses their activation (Xu et al., 2005). However, when unfolded/misfolded proteins accumulate, Grp78/BiP preferentially binds to unfolded/misfolded proteins, resulting in activation of the ER stress response, including an upregulation of genes encoding Grp78/BiP, protein disulfide isomerase (PDI) and down regulation of protein synthesis (Kaufman, 1999; Bertolotti et al., 2000). PERK activation induces the eukaryotic initiation factor 2 alpha subunit (eIF2α) kinase and phosphorylation of eIF2α resulting in inhibition of protein translation (Xu et al., 2005). Activation of IRE1α leads to the alternative splicing of the transcription factor X-box binding protein 1 (XBP1) to the spliced XBP1 form to induce genes that regulate protein quality control in the ER (Xu et al., 2005). Although ER stress is usually a short term homeostatic event essential for cell survival, prolonged and severe ER stress can trigger apoptosis by ER stress-specific cell death signals, including C/EBP homologous protein (CHOP) and caspase-12 (Nakagawa et al., 2000; Kaufman, 2002).

It has previously been shown that mutant SOD1 accumulates inside the ER, where it forms insoluble high molecular weight aggregates and interacts with Grp78/BiP in spinal cord microsomal fractions (Kikuchi et al., 2006). Markers of ER stress activation have been shown in spinal cord sections of ALS patients and in mouse models of ALS (Kikuchi et al., 2006; Atkin et al., 2008). Pathology studies show that ER stress is evident in spinal cords of ALS patients suggesting that ER stress-induced apoptosis may contribute to motor neuron death (Ilieva et al., 2007; Atkin et al., 2008). The ER stress response is also activated in mouse models of ALS, although the time course is controversial (Atkin et al., 2006, 2008; Kikuchi et al., 2006; Ilieva et al., 2007; Nishitoh et al., 2008; Saxena et al., 2009; Wang et al., 2011).

The targets of mutant SOD1-induced toxicity in ALS pathology are the motor neurons and the skeletal muscle that it innervates (Cleveland and Rothstein, 2001). While the primary focus has been on selective defects in the motor neuron causing muscle weakness and atrophy, it has been shown that muscle-restricted SOD1 mutations also recapitulate the hallmark signs of ALS, albeit at a slower rate of progression (Dobrowolny et al., 2008; Wong and Martin, 2010). Thus, it has been proposed that defects in skeletal muscle, leading to muscle cell dysfunction, also contribute to the motor neuron pathology via a “dying-back” phenomenon. It has previously been shown that early markers of muscle adaptation in the G93A*SOD1 mouse (i.e., by 49d, prior to atrophy) include metabolic enzymes, particularly down regulation of enzymes of oxidative metabolism and upregulation of enzymes of glycolytic metabolism and increases in proteins involved in protein synthesis (Capitanio et al., 2012). Putative markers of disease progression, which were altered at 98d when atrophy was evident, include glycolytic enzymes which decrease and cell stress markers (i.e., heat shock proteins) and transport proteins (i.e., albumin), which increase.

The intracellular mechanisms leading to altered gene/protein expression in skeletal muscle with disease-induced plasticity are not fully understood. However, there are reports of mitochondrial depolarization leading to reduced mitochondrial Ca^2+^ buffering and increased cytosolic Ca^2+^ which may trigger events in the muscle atrophy process (Zhou et al., 2010; Yi et al., 2011). Recently we reported impaired intracellular Ca^2+^ regulation in the sarcoplasmic reticulum (SR) in muscle fibers from G93A*SOD1 mice (Chin et al., 2014). The changes intracellular Ca^2+^ occurred prior to the decline in motor function (by 90d) and were associated with decreases in myocellular Ca^2+^ buffering proteins SERCA1, SERCA2 and parvalbumin. Based on the known association between SR/ER Ca^2+^ regulation and protein folding (Glembotski, 2012; Prell et al., 2013), and the putative contribution of skeletal muscle defects to the progression of ALS, we hypothesized that ER stress would be induced in skeletal muscle. Thus, the primary aim of this study was to investigate the ER stress signaling pathway in skeletal muscle at three different ages across the lifespan of the G93A*SOD1 mouse model of ALS. A secondary aim was to compare key markers of ER stress in skeletal muscles of varying fiber type composition and metabolic capacities as well as to non-muscle tissue. Our findings indicate that ER stress is activated in skeletal muscle of G93A*SOD1 mice as early as 70d, with ER stress pathways leading to inhibition of protein translation. Our data further suggest that defects in myocellular protein handling and activation of apoptosis may contribute to the muscle atrophy and weakness observed in ALS.

## Methods

### Ethics Statement

All procedures were conducted under a protocol approved by the Institutional Animal Care and Use Committee (IACUC) of the University of Maryland, College Park.

### Animals

Control C57BL/6 SJL hybrid female and transgenic ALS B6SJL-Tg(SOD1-G93A)1Gur/J (G93A*SOD1) male mice were obtained from The Jackson Laboratory. Wild-type control (WT) and transgenic G93A*SOD1 heterozygote (ALS-Tg) mice were bred to establish a colony at our animal care facility at the University of Maryland. Mice were weaned at postnatal day 21 and genotyped. Male and female ALS-Tg mice along with their wild-type littermates were investigated at a range of ages from the pre-symptomatic to the symptomatic stages of the disease: (i) early pre-symptomatic at postnatal day 70 (70d); (ii) late pre-symptomatic at postnatal day 90 (90d); and (iii) end stage at postnatal day 120–140 (120–140d) (see Supplementary Material Table S1). Early signs of disease such as muscle tremors can be detected between 65 and 90d but overt muscle weakness and limitations in mobility do not occur until 100–120d (Rosen et al., 1993). We chose the 70d and 90d time points based on differences observed in single muscle fiber resting intracellular Ca^2+^ concentration that we now have reported (Chin et al., 2014). The final time point (120–140d) was based on symptom progression, with the date of use determined by the inability of the mouse to right itself after 30 s of being placed on its side as previously described by others (Deforges et al., 2009).

At time of use, animals were euthanized by CO_2_ inhalation followed by cervical dislocation. Skeletal muscles, cardiac muscle, and liver were harvested, quickly frozen in liquid nitrogen and stored at −80°C for subsequent analysis. Various skeletal muscles were harvested in order to assess differences in ER stress between muscles of varying fiber type and of different oxidative and glycolytic capacities. White gastrocnemius (WG) has primarily fast glycolytic fibers (97% type IIB, 1.5% IIX/B and 1.5% IIX), red gastrocnemius (RG) primarily fast oxidative glycolytic fibers (22% type IIB, 3% IIX/B, 20% IIX, 42% IIA and 8% and type I) (Bloemberg and Quadrilatero, 2012) and diaphragm (DIA) has a mixed fiber type including both slow oxidative, fast oxidative and fast glycolytic fibers (39% type IIX, 23% type IIX/B, 23% IIA/X and 10% type I (Guido et al., 2010). Tibialis anterior (TA) has primarily fast glycolytic fibers (50% type IIB, 40% type IIX and 10% IIA), with reports of a fiber type shift to more oxidative (20% IIB, 10% IIX and 70% IIA) at 115d in the G93A*SOD1 mouse (Deforges et al., 2009). In WT mouse muscle, glycolytic capacity is greatest in type IIB > IIB/X > IIX = type I > IIA/X > IIA (Bloemberg and Quadrilatero, 2012) and thus expected to be highest in WG > TA > DIA > RG. Conversely, in WT, oxidative capacity is greatest in type IIA > type I = type IIX > IIB and thus would be highest in RG > DIA > TA > WG.

### Protein Extraction

The superficial (white) and deep (red) gastrocnemius, DIA, cardiac muscle, and liver tissues were used for assessment of protein levels using western blot technique. Tissue samples were homogenized on ice using a polytron at 50% maximum power for three 10 s bursts, separated by 30 s in ice cold lysis buffer (20 mM Hepes, pH = 7.5, 150 mM NaCl, 1.5 mM MgCl_2_, 0.1% Triton X-100, 20% Glycerol) containing 1 mM DTT and protease inhibitor cocktail (cOmplete mini EDTA-free Protease Inhibitor Cocktail, Roche). After 20 min of incubation at 4°C followed by centrifugation for 5 min at 20,000× g, the supernatant was collected, quick frozen in liquid nitrogen and stored at −80°C until required.

### Western Blot Analyses

Total protein concentration in the samples was determined using a BCA protein assay kit (Thermo Scientific). Samples were then prepared with loading buffer and denatured by incubating samples at 100°C for 5 min. For western blot analyses, 30 μg total protein was loaded on bis-acrylamide gels and separated using polyacrylamide gel electrophoresis (PAGE). Samples were then transferred to PVDF membrane (Millipore) and blocked with 5% (w/v) non-fat dry milk in Tris-buffered saline (pH 8.0) for 1 h. The appropriate primary antibodies were added: Grp78/BiP (BD Biosciences) (PERK, phospho-PERK (Thr980), IRE1α, eIF2α, phospho-eIF2α (Ser51), PDI, and CHOP; 1:1000, Cell Signaling Technology) and membranes were incubated at 4°C overnight, washed and then and subsequently probed with HRP-linked anti-rabbit IgG or anti-mouse IgG antibodies (1:1000, Cell Signaling Technology) 1 h at room temperature. Secondary antibodies were detected using HRP-linked chemiluminescence with SuperSignal West Dura Chemiluminescence Substrate (Thermo Scientific) and imaged using the chemiluminescence imaging system (GeneGnome, Syngene). The signal for the target protein of each sample was quantified using densitometry (Image J Software) and expressed in arbitrary unit (AU). GAPDH (1:2000, Thermo Scientific) or β-actin (1:1000, Cell Signaling Technology) was used to confirm equal protein loading across samples.

### Mass Spectrometry based Protein Relative Quantification

To confirm differential expression of ER stress proteins using a non-antibody based method, we completed in-gel digestion of proteins in the molecular weight range of the Grp78/BiP protein. We focused on the Grp78/BiP protein based on preliminary work with an antibody that gave us a divergent response (Chen et al., 2012) to the one we report here. Skeletal muscle total protein samples were prepared as described previously. For the purpose of protein separation, 30 μg of total protein was loaded onto 8% one-dimensional SDS-PAGE gel. After gel electrophoresis, protein bands were stained using a protein blue stain kit (Thermo Scientific). The targeted bands (~80 kDa) were carefully excised and in-gel tryptic digestion carried out following standard procedure. Briefly, proteins were reduced with 5 mM DTT, alkylated with 55 mM iodoacetamide, and digested with 20 ng/μL trypsin (Life Technologies™) at 37°C overnight. All reagents were dissolved in 50 mM ammonium bicarbonate (pH 8.5).

After trypsin digestion, peptide products were collected and analyzed by nano LC-MS/MS analysis using LTQ Orbitrap mass spectrometer coupled to a Shimadzu 2D Nano HPLC system. Peptides were loaded with an autosampler into an Zorbax SB-C18 trap column (0.3 × 5.0 mm) (Agilent Technologies, Palo Alto, CA, USA) at 10 μL/min with solvent A (97.5% water, 2.5% ACN, 0.1% formic acid) for 10 min, then eluted and separated at 300 nL/min with a gradient of 0–35% solvent B (2.5% water, 97.5% ACN, 0.1% formic acid) in 30 min using a Zorbax SB-C18 nano column (0.075 × 150 mm). The mass spectrometer was set to acquire a full scan at resolution 60,000 (m/z 400) followed by data dependent MSMS analysis of top 10 peaks with more than one charge in the linear ion trap at unit mass resolution. The resulting LC-MS/MS data were searched against a mouse protein database generated from uniprot and a common contaminant database using Mascot (v2.3) and Sequest search engines through Proteome Discoverer (v1.4). Carbomidomethylation at Cys was set as fixed modification. Methionine oxidation and asparagine and glutamine deamidation were set as variable modification. Spectral counting with normalized total spectra was carried out using Scaffold software, (Proteome Software, Inc). Protein probability >99% and at least one unique peptide with a probability score >95% were set to as minimum requirement for protein identification.

### Gene Expression

In order to investigate transcriptional events involved in ER stress pathway, we isolated mRNA from TA muscle (TA) and examined transcript levels of XBP-1, GRP78/BiP, and CHOP. Briefly, total RNA was isolated using TriPure Reagent (Roche) and RNA content was determined by using a NanoDrop spectrophotometer and mRNA was diluted to 5 ng/μL. Reverse transcription from mRNA to cDNA was conducted by using One-Step RT-PCR System (Life Technologies™). Semi-quantitative PCR (sqPCR) was used to determine gene transcriptional levels and the primer information such as XBP-1, CHOP, and 18 s was acquired from a previous study (Rosen et al., 1993). The band intensity of PCR products were quantified using densitometry (Image J Software) and expressed in arbitrary unit (AU). For XBP-1, two variants of XBP1 mRNA are expressed in cells. Under normal conditions, un-spliced XBP1 mRNA (XBP1-u) is expressed. However, as ER stress is induced, a spliced form of XBP1 mRNA (XPB1-s) will be expressed. Thus, upregulation of XBP1-s mRNA is a marker of ER stress activation downstream of IRE1α (Wu et al., 2011).

### Data Analysis

To determine statistical differences in protein and mRNA expression level between genotype (WT vs. ALS-Tg) and Age (70d, 90d and 120–140d) data were analyzed using two-way ANOVAs. Where interaction effects (genotype × age) were observed, the main effects are not indicated in the Results section but can be found in the Supplementary material (Table S1). For significant interaction effects, Tukey *post hoc* tests were used to determine differences across time points for ALS-Tg (i.e., 70d vs. 90d). *T*-tests were used to determine differences between WT and ALS-Tg at each time point. Statistical significance was accepted as *p* < 0.05.

## Results

### ER Stress Pathway is Induced in Skeletal Muscle of ALS Mice

**Figure 1:**
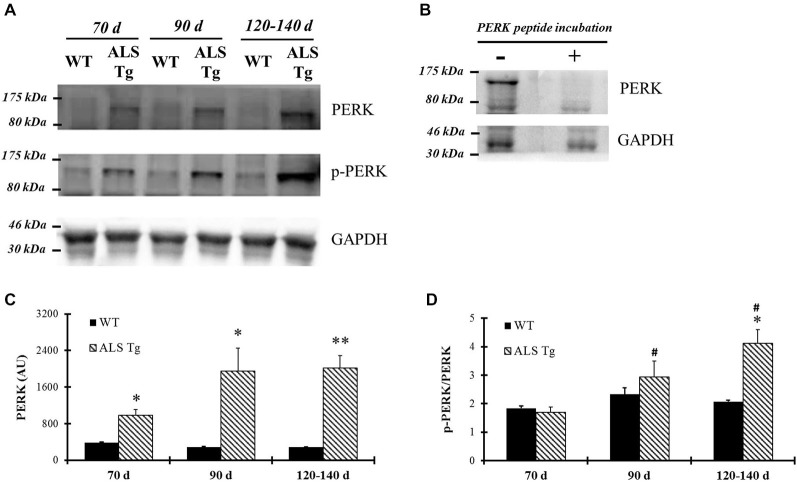
PERK and phospho-PERK are up-regulated in skeletal muscle of G93A*SOD1 amyotrophic lateral sclerosis (ALS)-Tg mice. (A) White gastrocnemius (WG) muscle tissues from different ages of wild-type (WT) and transgenic G93A*SOD1 (ALS-Tg) mice were collected to determine protein expression levels using western blot technique. Primary antibodies PERK, phospho-PERK, GAPDH were used and representative images are shown. Three postnatal ages were examined as follows: early pre-symptomatic (70d; *n* = 3 each for WT and ALS-Tg), late pre-symptomatic (90d; *n* = 5 each for WT and ALS-Tg), and symptomatic (120–140d; *n* = 3 each for WT and ALS-Tg) mice. **(B)** Protein samples were incubated either with or without PERK peptides and then PERK protein levels were detected using western blots technique. **(C)** Analysis of average arbitrary units (AU) obtained by densitometry for PERK. **(D)** Analysis of average ratio of phospho-PERK to total PERK. Data in **(C)** and **(D)** are presented as mean ± S.E; *,*p* < 0.05; **,*p* < 0.01 WT vs. ALS-Tg in the same age group. ^#^*p* < 0.05 vs. 70d.

**Figure 2:**
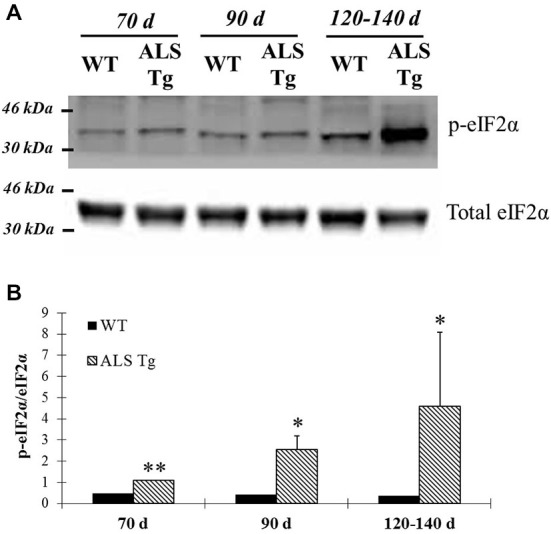
Phosphorylation of eIF2α is up-regulated in skeletal muscle of G93A*SOD1 ALS-Tg mice. (A) Total protein from WG muscle tissues was isolated from wild-type (WT) and transgenic G93A*SOD1 (ALS-Tg) mice. Western blots showed phospho-eIF2α (Ser51) and total eIF2α. Three different disease stages were used: early pre-symptomatic (70d; *n* = 3 each for WT and ALS-Tg), late pre-symptomatic (90d; *n* = 5 each for WT and ALS-Tg), and symptomatic (120–140d; *n* = 3 each for WT and ALS-Tg) mice. **(B)** Analysis of ratio of phospho-eIF2α to total eIF2α. Data in **(B)** is presented as mean ± S.E; *,*p* < 0.05; **,*p* < 0.01 WT vs. ALS-Tg in the same age group.

PERK and IRE1α are two ER stress sensors known to be upregulated when ER stress is induced. In our study, PERK protein level was upregulated 2.6-fold in WG muscle of ALS-Tg vs. WT mice at 70d (*p* = 0.01), 5.4-fold at 90d (*p* = 0.025) and 5.2-fold at 120–140d (*p* = 0.001; Figures 1A,C). There was no difference in PERK level of ALS-Tg WG between 70, 90 and 120–140d (no main effect for age or genotype × age interaction). To assess the specificity of the antibody for total PERK, the antibody was pre-incubated with a PERK-antibody blocking peptide. Under these conditions, the protein band at ~140kDa identified as PERK was not visible (Figure 1B), confirming the specificity of the PERK antibody. Since activated PERK undergoes auto-phosphorylation, we also assessed the ratio of phospho-PERK to total PERK. The phospho-PERK/total PERK ratio was increased 2-fold in WG of ALS-Tg mice at 120–140d (*p* = 0.012) indicating greater activation of existing PERK protein at the symptomatic age (Figures 1A,D). Previous studies have shown that PERK can activate eIF2α kinase, resulting in phosphorylation of eIF2α at Ser51 and suppression of protein synthesis during ER stress (Wu et al., 2011). We therefore assessed the downstream effects of activation of PERK. In WG muscle, the ratio of phospho-eIF2α/total eIF2α was increased 2.3-fold in ALS-Tg vs. WT at 70d (*p* = 0.005), remained elevated at 90d (*p* = 0.048) and increased further to 12-fold at 120–140d (*p* = 0.011; Figures 2A,B). For phospho-eIF2α/total eIF2α there was a genotype × age interaction effects with the increase in ALS-Tg only increasing between 70d and 120–140d (70d vs. 90d; *p* = 0.227; 70d vs. 120–140d *p* = 0.018; 90d vs. 120–140 *p* = 0.062). Total eIF2α was not altered, just phosphorylation at Ser51, indicating inhibition of protein translation at these ages.

**Figure 3:**
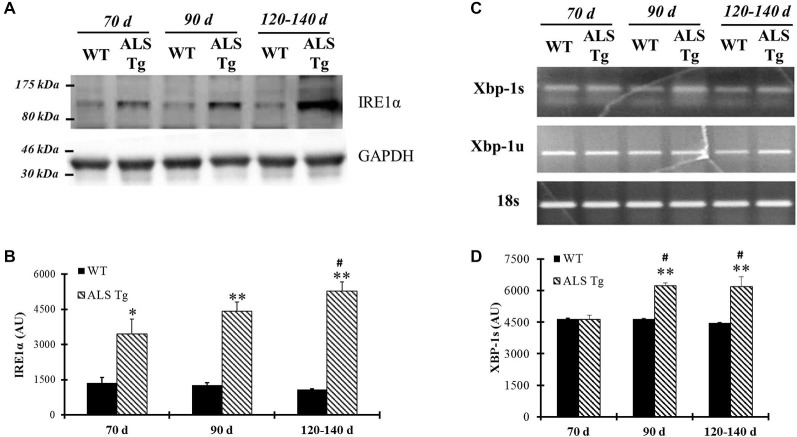
IRE1α protein level and spliced Xbp-1 transcriptional level are up-regulated in skeletal muscle of G93A*SOD1 ALS-Tg mice. (A) Protein was isolated from WG muscle of different ages of wild-type (WT) and transgenic G93A*SOD1 (ALS-Tg) mice and western blot was performed by using antibody specific for IRE1α. GAPDH was used as the total protein loading control. Three postnatal ages were examined as follows: early pre-symptomatic (70d; *n* = 3 each for WT and ALS-Tg), late pre-symptomatic (90d; *n* = 5 each for WT and ALS-Tg), and symptomatic (120–140d; *n* = 3 each for WT and ALS-Tg) mice. **(B)** Analysis of average arbitrary units (AU) obtained by densitometry for IRE1α. **(C)** Total mRNA was isolated by using Tibialis anterior (TA) muscle tissues and un-spliced (Xbp-1u) and spliced Xbp-1 (Xbp-1s) transcriptional levels were determined by using semi-quantitative PCR and PCR results are shown by running the products in 1.5% agarose gel. 18S was used as the internal control. Three different ages animals as mentioned previously were used. **(D)** Analysis of average arbitrary units (AU) obtained by densitometry for Xbp-1s. Data in **(B,D)** are presented as mean ± S.E; *,*p* < 0.05; **,*p* < 0.01 WT vs. ALS-Tg in the same age group. ^#^*p* < 0.05 vs. 70d.

In addition to PERK, up-regulation of ER stress sensor IRE1α was also observed in WG of ALS-Tg mice by 70d. IRE1α protein levels were increased 2.5-fold at 70d (*p* = 0.043) remained elevated at 90d (*p* = 0.0005) and showed a further increase to 4.9-fold WT levels at 120–140d (*p* = 0.0002 vs. WT by *t*-test; *p* = 0.008 for 70d vs. 120–140d for ALS-Tg by Tukey *post hoc*; Figures 3A,B). XBP1 mRNA splicing is commonly used to indicate upregulation of IRE1α since activation of IRE1α leads to mRNA splicing. Thus, we investigated transcript levels of the un-spliced (XBP-1u) and spliced XBP1 (XBP-1s) forms of XBP-1. In TA muscle, XBP-1s mRNA was increased to 1.3- and 1.4-fold at 90d (*p* = 0.001) and 120–140d (*p* < 0.0001). There was a genotype × age interaction effect with XBP1-s being higher at 90d vs. 70d (*p* = 0.002) and at 120–140d vs. 70d (*p* = 0.002). XBP1-u mRNA was not altered (Figures 3C,D).

**Figure 4:**
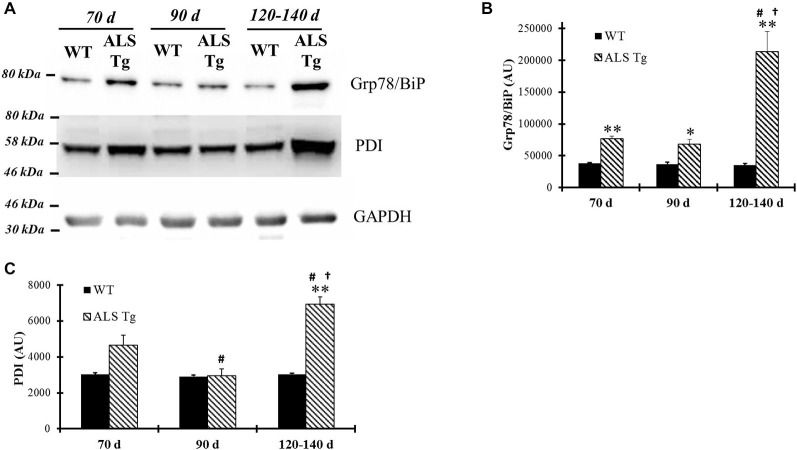
ER chaperones Grp78/BiP and protein disulfide isomerase (PDI) are up-regulated in skeletal muscle of G93A*SOD1 ALS-Tg mice. (A) WG muscle tissues were used to determine Grp78/BiP and PDI expressions using western blot technique from different ages of wild-type (WT) and transgenic G93A*SOD1 (ALS-Tg) mice. Representative images of Grp78/BiP and PDI are shown. Three postnatal ages were examined as follows: early pre-symptomatic (70d; *n* = 3 each for WT and ALS-Tg), late pre-symptomatic (90d; *n* = 5 each for WT and ALS-Tg), and symptomatic (120–140d; *n* = 3 each for WT and ALS-Tg) mice. **(B,C)** Analysis of average arbitrary units (AU) obtained by densitometry of PDI. Data in B are presented as mean ± S.E; **,*p* < 0.01 WT vs. ALS-Tg in the same age group. ^#^*p* < 0.05 vs. 70d and ^†^*p* < 0.05 vs. 90d.

Since cellular stress results in induction of ER chaperone proteins to handle misfolded and unfolded proteins, we examined changes in PDI and Grp78/BiP protein levels, two proteins involved in post-translational modification and known to be up-regulated with ER stress activation (Xu et al., 2005). Grp78/BiP was upregulated 2-fold in WG of ALS-Tg vs. WT mice at 70d (*p* = 0.006), remained elevated at 90d (*p* = 0.025) and increased further to 6.7-fold at 120–140d (*p* = 0.005) (Figures 4A,B). There was a genotype × age interaction effect with Grp78/BiP in ALS-Tg being different at 70d vs. 120–140d (*p* < 0.001) and at 90d vs. 120–140d (*p* < 0.001). Increased expression of PDI was also observed (2.2-fold but only at 120–140d (*p* = 0.001; Figures 4A,C). There was a genotype × age interaction effect for PDI with differences across all age groups for ALS-Tg (70d vs. 90d, *p* = 0.004; 70d vs. 120–140d, *p* = 0.001; 90d vs. 120–140d, *p* < 0.001). Taken together, these data show evidence of ER stress in skeletal muscle as early as 70d and further augmented at the symptomatic age in ALS-Tg mice.

**Figure 5:**
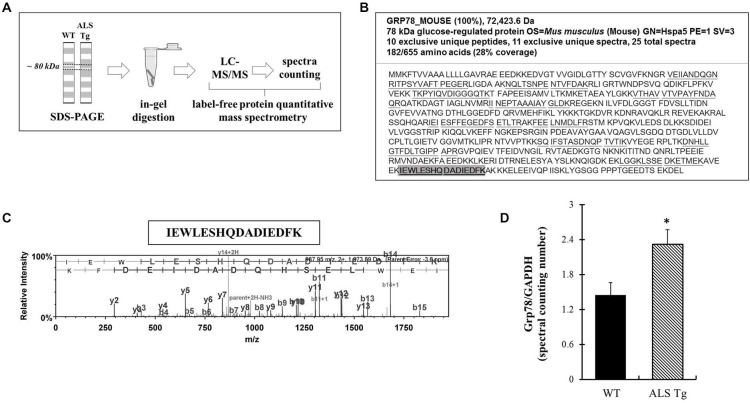
**Grp78/BiP protein identification and quantitation using label-free spectral counting-based mass spectrometry. (A)** Workflow of label-free spectral counting-based protein quantitative analysis using LC-MS/MS. Protein samples were separated using SDS-polyacrylamide gel electrophoresis (PAGE) and gel pieces excised at ~80 kDa for the purpose of in-gel trypsin digestion and LC-MS/MS analysis. Protein quantitative data analysis was conducted using spectral counting and interpreted by normalized total spectra numbers. **(B)** Grp78/BiP protein identification and peptide coverage using LC-MS/MS. **(C)** Representative mass-to-charge ratio spectrum and b-/y ions fragmentation of Grp78/BiP peptide (highlighted in **B**). **(D)** Protein quantitative data analysis using the ratio of normalized total spectra numbers of Grp78/BiP to a house keeping protein GAPDH. Three independent muscles from 120–140d old wild type (WT) and ALS-Tg mice were analyzed. * *p* < 0.05, WT vs. ALS-Tg.

To confirm antibody-based findings of the Grp78/BiP increase in skeletal muscle of ALS-Tg mice, we carried out relative protein quantification using LCMSMS and spectral counting compare Grp78/BiP protein levels between genotypes (Figure 5A). Grp78/BiP protein was identified by 11 exclusive unique spectra which contributed to the identification of 10 exclusive unique Grp78/BiP peptides (Figures 5B,C). Protein quantitative data analysis showed that Grp78/BiP was more abundant in skeletal muscle of ALS mice as spectral counting numbers were significantly higher in ALS-Tg vs. WT mice (Figure 5D). Collectively, our label-free spectral counting-based protein quantitative data is consistent with western blot data, supporting our notion that ER stress is activated in skeletal muscle of ALS mice.

### ER Stress-Specific Cell Death Signal is Induced in Skeletal Muscle of ALS Mice

**Figure 6:**
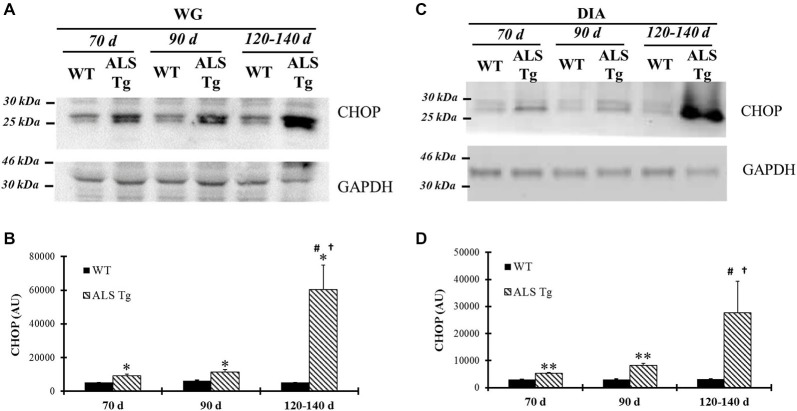
CHOP is up-regulated in skeletal muscles of G93A*SOD1 ALS-Tg mice. (A) WG muscle tissues were obtained from wild-type (WT) and transgenic G93A*SOD1 (ALS-Tg) mice of different ages and used to determine CHOP protein levels using western blot technique. GAPDH was used as the total protein loading control. Three postnatal ages were examined as follows: early pre-symptomatic (70d; *n* = 3 each for WT and ALS-Tg), late pre-symptomatic (90d; *n* = 5 each for WT and ALS-Tg), and symptomatic (120–140d; *n* = 3 each for WT and ALS-Tg) mice. **(B)** Analysis of average arbitrary units (AU) obtained by densitometry of CHOP in WG. **(C)** Same as **(A)**, with diaphragm (DIA) muscle tissues used to assess CHOP protein level. **(D)** Analysis of average arbitrary units (AU) obtained by densitometry of CHOP in DIA. Data in **(B,D)** are presented as mean ± S.E; *,*p* < 0.05; **,*p* < 0.01 WT vs. ALS-Tg in the same age group. ^#^*p* < 0.05 vs. 70d and ^†^*p* < 0.05 vs. 90d.

Several mechanisms have been suggested to link the ER stress pathway to cell death, including activation of the ER stress-specific cell death signal CHOP (Xu et al., 2005). In WG of ALS-Tg mice, CHOP was upregulated 1.8-fold at 70d (*p* = 0.041), remained elevated at 90d (*p* = 0.025) and further increased to 12-fold at 120–140d (*p* = 0.019; Figures 6A,B). There was a significant genotype × age interaction effects with ALS-Tg only being different at 70d vs. 120–140d (*p* < 0.001) and at 90d vs. 120–140d (*p* < 0.001). There were no changes in CHOP mRNA (data not shown), indicating that there is post-translational modification and increased stability of CHOP protein (Ohoka et al., 2007). In addition to evaluating CHOP induction in the limb muscle, we also investigated DIA muscle since atrophy of this muscle results in respiratory failure and death in ALS mice (Tankersley et al., 2007). In DIA, CHOP protein expression was increased 1.7-fold in ALS-Tg vs. WT mice at 70d (*p* = 0.008), remained elevated at 90d (*p* = 0.001) and then increased further to 9-fold at 120–140d but due to the high degree of variability in CHOP elevation, ALS-Tg vs. WT was not significant at 120–140d (*p* = 0.10), there was a genotype × age interaction effect with CHOP showing an increase in ALS-Tg at 120–140d vs. 70d (*p* = 0.004) and 120–140d vs. 90d (*p* = 0.005) (Figures 6C,D).

### Greater Activation of ER Stress Pathway in Glycolytic vs. Oxidative Muscle of ALS Mice

**Figure 7:**
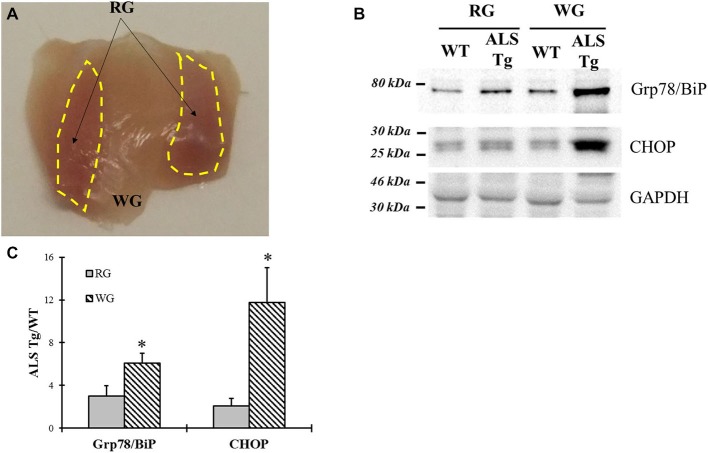
Comparison of Grp78/BiP and CHOP protein levels between white and red gastrocnemius (RG) muscle tissues of G93A*SOD1 ALS-Tg mice. (A) Image of deep portion of gastrocnemius muscle showing white and RG (yellow dashed areas) muscle region. **(B)** White (WG) and red (RG) gastrocnemius muscle tissues of symptomatic animals (120–140d; *n* = 3 each for WT and ALS-Tg) were collected. Grp78/BiP and CHOP protein levels were determined using western blot technique.** (C)** Analysis of Grp78/BiP and CHOP average ratio of ALS-Tg to WT. Data in **(B)** is presented as mean ± S.E; *,*p* < 0.05; RG vs. WG.

On dissection we noted that the superficial gastrocnemius muscle was more red than white in the ALS-Tg mice (Figure 7A). Previous studies showed that fast type IIb motor units are affected first during ALS disease progression in the G93A*SOD1 mouse (Hegedus et al., 2009). Thus, we wanted to determine whether ER stress is activated to a greater extent in fast glycolytic vs. fast oxidative skeletal muscle by comparing two typical ER stress markers Grp78/BiP and CHOP between WG (fast glycolytic) and RG (fast oxidative) muscle. At the symptomatic age (120–140d), both Grp78/BiP and CHOP were induced to greater extent in WG vs. RG (2.0- and 5.6-fold, respectively; *p* < 0.05; Figures 7B,C), indicating that ER stress activation is greater in fast glycolytic muscle.

### ER Stress Markers are not Induced in Cardiac Muscle and Liver Tissues of ALS Mice

**Figure 8:**
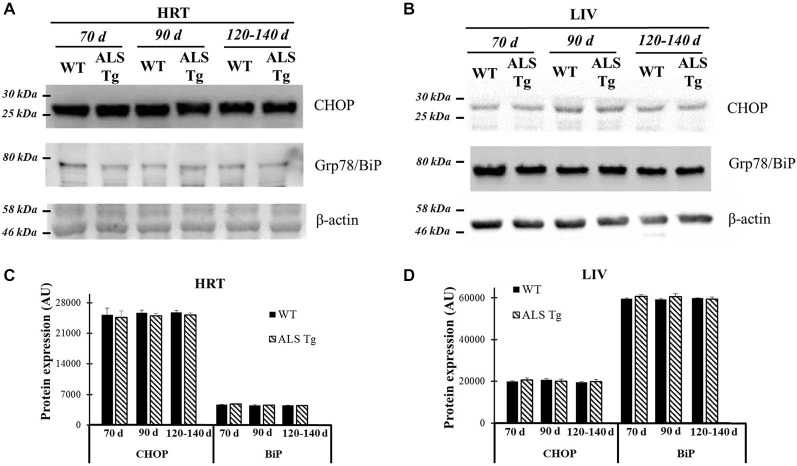
Grp78/BiP and CHOP protein levels in cardiac muscle and liver tissue of G93A*SOD1 ALS-Tg mice. (A) Cardiac muscle (HRT) was collected and protein levels determined using western blot technique. Grp78/BiP and CHOP antibodies were used and three postnatal ages were examined: early pre-symptomatic (70d; *n* = 3 each for WT and ALS-Tg), late pre-symptomatic (90d; *n* = 5 each for WT and ALS-Tg), and symptomatic (120–140d; *n* = 3 each for WT and ALS-Tg) mice. **(B)** Liver tissues (LIV) was collected and protein levels were determined as described above. Analysis of average arbitrary units (AU) obtained by densitometry of Grp78/BiP and CHOP in HRT **(C)** and in LIV** (D)**. Data in **(C,D)** are presented as mean ± S.E.

ER stress is activated when misfolded proteins accumulate in the ER lumen. One could argue that the ER stress activation we observed in our study may be a non-skeletal muscle specific event since the animal model we used is a whole-body SOD1 protein mutation and accumulation of mutant SOD1 could activate ER stress in all tissues, including skeletal muscle. Thus, we investigated the ER stress pathway in non-pathological tissues such as cardiac muscle and liver. Two classical ER stress markers, Grp78/BiP and CHOP, were not different between WT and ALS-Tg mice for heart or liver at any age (see Figure 8). Therefore, ER stress activation is observed in skeletal but not cardiac muscle or other highly oxidative tissues like liver in ALS mice.

## Discussion

In this study we show that ER stress is activated in skeletal muscle of G93A*SOD1 mice and thus may play a role in muscle atrophy in ALS. This is based on evidence that: (i) ER stress is activated at 70d, an early pre-symptomatic age and is further upregulated at 120–140d, an age when mice are symptomatic; (ii) skeletal muscle ER stress induces the cell death signal CHOP; (iii) ER stress is activated to a greater extent in highly glycolytic muscles with primarily type IIb fibers which are affected by an early loss of fast fatigable motor axons; and (iv) the ER stress activation is specific to skeletal vs. cardiac muscle. These data support the hypothesis that ER stress plays a role in muscle atrophy in ALS mice.

### ER Stress Response in ALS

**Figure 9:**
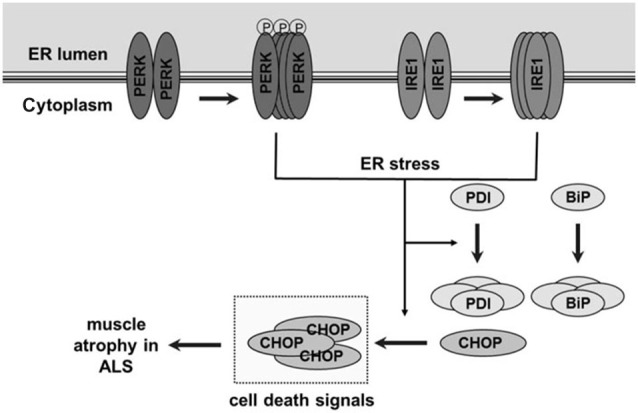
**Schematic figure showing unfolded protein response and endoplasmic reticulum (ER) stress pathway and its proposed role in skeletal muscle atrophy and weakness in ALS**. In skeletal muscle of ALS-Tg mice, the G93A*SOD1 mutation leads to oxidative stress and protein misfolding. This leads to an age-dependent activation of ER stress sensors protein kinase RNA-activated-like ER kinase (PERK) and inositol-requiring kinase 1-alpha (IRE1α). Normally, these ER stress sensors physically interact with the ER chaperone immunoglobulin binding protein (Grp78/BiP) which suppresses their activation but accumulation of unfolded/misfolded proteins activates Grp78/BiP, including an upregulation of Grp78/BiP and PDI protein expression. Prolonged and severe ER stress can trigger apoptosis by ER stress-specific cell death signals, including C/EBP homologous protein (CHOP) and caspase-12, leading to muscle atrophy.

Our lab previously reported impairments in SR Ca^2+^ uptake, leading to elevations in resting cytosolic Ca^2+^ concentration in muscle fibers from G93A*SOD1 mice (Chin et al., 2014). We therefore hypothesized that altered intracellular Ca^2+^ in association with increased oxidative stress in muscle cells would lead to misfolded proteins and activate the UPR and ER stress responses. We propose a model (Figure 9) where age-dependent activation of ER stress sensors PERK and IRE1α in response to misfolded proteins leads to increases in protein chaperones Grp78/BiP and PDI in skeletal muscle. Prolonged oxidative stress (in this case due to mutant SOD1) and persistent accumulation of misfolded proteins further augments the ER stress response and activates the apoptotic signal CHOP leading to muscle atrophy. At 70d, an age where muscle grip function is still 100% of WT levels (Chin et al., 2014), there is already an ~2-fold increase in the ER stress markers PERK, IRE1α, Grp78/BiP and CHOP. By 84d grip function is reduced to 77% WT levels and by 120–140d, when grip function is zero (i.e., mice are no longer able to grasp a metal grid) (Chin et al., 2014), there are further increases in IRE1α, Grp78/BiP and CHOP as well as increased phospho-PERK and phospho-eIF2α/total eIF2α. Based on these data we cannot determine if impaired Ca^2+^ regulation and ER stress are causative or a consequence of muscle atrophy, but only that they are associated. Using the same animal model, studies using MRI to asses muscle volume show significant muscle loss as early as 8 weeks of age and continuous loss over the lifespan of these mice (Marcuzzo et al., 2011; Mead et al., 2011). Collectively these data show muscle atrophy and weakness over the time frame that we observed increases in markers of ER stress, suggestive of some involvement of protein misfolding, activation of the ER stress response and possibly apoptosis. The impairment in muscle protein translation as well as apoptosis would contribute to muscle cell atrophy and weakness which, in conjunction with motoneuron degeneration, would contribute to the pathophysiology and disease progression in ALS.

Proteins that require folding and post-translational modification, primarily secretory and membrane bound proteins, are processed in the ER. Proteins that do not fold properly are degraded by the ubiquitin proteasome pathway. If misfolded/unfolded proteins accumulate, the UPR is triggered to promote proper folding and autophagy is activated to support cell survival. However, if there is a persistent increase in misfolded/unfolded proteins that exceeds the capacity of the ER to regulate proper folding, then protein translation is inhibited to reduce protein load and there is activation of an ER stress-induced cell death pathway via upregulation of CHOP. Activation of the ER stress response is increasingly being recognized as a cellular mechanism in neurodegenerative as well as metabolic diseases such as diabetes (Ozawa et al., 2005; Chambers and Marciniak, 2014). The biological role of ER stress activation in motor neurons has been shown in one study in which deletion of an ER stress-induced pro-apoptotic signaling protein (puma) in ALS mice resulted in improved motor neuron survival and delayed disease onset and motor dysfunction (Kieran et al., 2007). Although our study did not directly investigate the cellular consequences of ER stress activation in skeletal muscle of ALS mice, it is the first study to show the associated changes in ER stress markers in skeletal muscles across the lifespan of the G93A*SOD1 mouse.

Other studies with both ALS patients and transgenic G93A*SOD1 mice support the role of ER stress-related cellular dysfunction in ALS pathophysiology (Atkin et al., 2006, 2008; Kikuchi et al., 2006; Ilieva et al., 2007; Nishitoh et al., 2008; Saxena et al., 2009; Wang et al., 2011). Studies examining lumbar spinal cord sections of G93A*SOD1 mice, demonstrated significant upregulation of three ER stress sensors, PERK, IRE1α and ATF-6 as early as 60d, a time point at which these mice do not show any symptoms, suggesting they may trigger disease pathophysiology (Atkin et al., 2008). In addition, the ER stress-specific cell death markers, CHOP and caspase-12, were activated, indicating apoptosis was induced before symptom onset (Atkin et al., 2008). ATF6, IRE1α, CHOP, and caspase-12 were also shown to be up-regulated in the lumbar spinal cord sections of G93A*SOD1 mice at 90d, at a late pre-symptomatic stage of the disease (Atkin et al., 2006). In yet other studies, a broad ER stress response occurred in spinal cords only in the end stage of ALS (~140d) when the mice were symptomatic which does not support the role of ER stress in ALS pathology (Kikuchi et al., 2006). Levels of ER stress-related proteins including PERK, IRE1α, ATF6, XBP-1, Grp78/BiP, and CHOP were upregulated in motor neurons of the spinal cords of ALS patients (Atkin et al., 2008). However, evidence from these studies is based on the association of ER stress markers with disease presence.

### Impaired Protein Translation in Muscle Atrophy and ALS

Motor neurons and skeletal muscles are two targets of mutant SOD1-mediated toxicity in ALS. In skeletal muscle cells, ER stress leading to inhibition of protein translation and decreased protein synthesis could explain the reduction in muscle mass of the G93A*SOD1 mouse (Gurney et al., 1994; Marcuzzo et al., 2011; Mead et al., 2011). We observed increases in PERK protein-kinase activity and increased phosphorylation of eIF2α on serine residue 51. Increased phospho-eIF2α has been shown to inhibit translation of messenger RNA into protein, effectively decreasing the protein load (Harding et al., 1999; Scheuner et al., 2001). Inhibition of protein translation can alter both muscle protein synthesis during growth but also muscle plasticity in disease. We recently reported alterations in intracellular Ca^2+^ levels in skeletal muscle of the G93A*SOD1 mice and reductions in the Ca^2+^ buffering proteins SERCA1, SERCA2 and parvalbumin (Chin et al., 2014). SERCA1 is the SR/ER Ca^2+^ ATPase isoform expressed in fast glycolytic fibers and SERCA2 is expressed in fast oxidative and slow fibers (Periasamy and Kalyanasundaram, 2007). Due to the shift to more oxidative fibers in WG (Hegedus et al., 2008) and TA (Deforges et al., 2009), we expected an increase in SERCA2 protein expression. However, SERCA2 protein was also decreased in skeletal muscle of ALS-Tg mice (Chin et al., 2014) despite a compensatory upregulation of SERCA2 mRNA (unpublished data). Thus, at least for SERCA2, we have observed transcriptional upregulation with no concurrent increase in protein translation. Our current data showing increased phospho-eIF2α/total eIF2α are consistent with this inhibition of protein translation as early as 70d. While there is an overall decrease in protein translation, this will specifically affect the secretory and transmembrane proteins that are processed in the ER (Chambers and Marciniak, 2014) such as the SERCA pump proteins. However, the muscle is still capable of increased protein synthesis and can upregulate expression of the required ER stress proteins. Future studies will be required to identify other muscle-specific proteins that are misfolded and that may contribute to atrophy and/or disease pathophysiology in ALS.

### ER Stress, Muscle Metabolic Capacity and Disease Pathology

There are limited reports about ER stress pathways in skeletal muscles of motoneuron disease, although there are clinical reports of ER stress and UPR being activated in inclusion body myositis (Vattemi et al., 2004; Nogalska et al., 2006), autoimmune myositis (Nagaraju et al., 2005; Vitadello et al., 2010), and myotonic dystrophy (Ikezoe et al., 2007). It has also been shown that ER stress response proteins IRE1α, PDI, and other ER chaperones are upregulated in skeletal muscle of mice in response to high fat diet feeding which lead to a decrease in protein synthesis and insulin resistance (Deldicque et al., 2010). ER stress sensors and ER chaperones have also been shown to be upregulated in skeletal muscle following exercise (Kim et al., 2011; Wu et al., 2011). Our study is consistent with these prior reports of ER stress response in diseases and under conditions of various cellular stresses such as altered metabolic substrate supply and changes in metabolic activity. Interestingly, our observation that ER stress was greater in WG compared to RG is consistent with the notion that an energetic stress contributes to activation of the ER stress response as glycolytic fibers are more likely to experience periods of hypoxia or perturbed energy homeostasis.

Previous studies have shown that glycolytic skeletal muscle is more susceptible to atrophy in response to hypoxia (de Theije et al., 2015) and in disease states such as cancer cachexia (Yu et al., 2008; Baltgalvis et al., 2009) and heart failure (Li et al., 2007). The DIA, a muscle of mixed fiber type with high glycolytic capacity, had levels of ER stress comparable to the glycolytic WG. It thus appears that metabolic capacity rather than muscle fiber type *per se* (i.e., fast vs. slow contracting) is a crucial determinant of susceptibility to ER stress in skeletal muscle cells. Muscles with a higher oxidative capacity have an increased capacity to respond to oxidative stress (Yu et al., 2008) and induce heat shock proteins (i.e., hsp70) (Locke and Tanguay, 1996; Tarricone et al., 2008). This may render protection and provide a greater capacity to refold proteins in highly oxidative muscle fibers. We speculate that skeletal muscles with high glycolytic capacity and those with high repetitive use (i.e., DIA) are most susceptible to ER stress due to the greater metabolic stress with contractile activity and a reduced capacity to deal with misfolded proteins.

### Potential Limitations

In the current study we have assessed markers of ER stress using commercially available antibodies. The specificity and selectivity of antibodies for the protein ligand are critical to the interpretation of data from western blot analyses. We have confirmed the specificity of some of these protein markers using either a blocking peptide (PERK) or mass spectrometry (Grp78/BiP). Supporting our data, a recent study using proteomics and bioinformatics tools reported activation of stress responses in gastrocnemius muscle of ALS-Tg mice at 98d including Alpha-crystallin B chain (Cryab), Heat shock protein HSP 90-beta (Hsp90ab1) and protein disulfide-isomerase A3 (Pdia3) that suggest abnormalities in the ER protein folding machinery and activation of the UPR (Capitanio et al., 2012). Our observations of increased expression of proteins involved in UPR and ER stress are consistent with this report and illustrate that two independent techniques have been used to confirm the differential expression of ER stress protein markers in skeletal muscle.

### Mutant SOD1, Mitochondrial Disruption and ER/SR Dysfunction

Previous studies indicated that mutant SOD1 is present within the ER lumen which may account for activation of ER stress in SOD1-linked ALS cases (Karch et al., 2009). Mutant SOD1 also accumulates in mitochondria specifically in motoneurons (vs. sensory neurons) (Sotelo-Silveira et al., 2009). Based on the ubiquitous expression of SOD1 (Hirano, 1991) one would expect activation of the UPR and ER stress pathway from mutant SOD1 in all tissues. However, our results of two typical ER stress markers, Grp78/BiP and CHOP, showed that ER stress is not present in cardiac muscles or liver tissues. We speculate that this is due to the high oxidative capacity, and increased mitochondrial content in these tissues. Motoneurons and glycolytic skeletal muscle, however, have low mitochondrial content and thus mitochondrial impairment will be detrimental to survival of these cells. Decreased mitochondrial inner membrane potential and mitochondrial Ca^2+^ buffering have been observed in response to osmotic stress and plasma membrane depolarization in muscle fibers of G93A*SOD1 mice (Yi et al., 2011). The same group has shown that reduced mitochondrial membrane potential is greatest in muscle fibers at the neuromuscular junction region (Zhou et al., 2010). Thus, changes in mitochondrial function leading to elevated cellular Ca^2+^ are specifically impaired at the skeletal muscle membrane nearest to its point of innervation. This may play an important role in the axonopathy associated with ALS (Fischer et al., 2004; Wong and Martin, 2010).

Vulnerable motoneurons also have reduced cytosolic Ca^2+^ buffering capacity and disrupted mitochondrial and ER Ca^2+^ buffering. Motoneurons with mutant (G93A) SOD1- had reduced mitochondrial membrane potential, lower ER Ca^2+^ release in response to a SERCA inhibitor and impaired mitochondrial Ca^2+^ buffering (Jaiswal and Keller, 2009; Jaiswal et al., 2009). Thus, impairment of mitochondrial and ER Ca^2+^ storage is thought to be central to motoneuron degenerative changes. Others have shown that mutant SOD1 aggregates with voltage dependent anion channels in mitochondria, specifically of motoneurons (Israelson et al., 2010). In the latter study, ADP transport but not Ca^2+^ uptake by motoneuron mitochondria was diminished. There is emerging data on the importance of Ca^2+^ regulation, mitochondrial function and ER protein misfolding in various diseases (Jaiswal, 2013; Prell et al., 2013), but the tissue-specific mechanisms altered leading to muscle atrophy and weakness are still not clear. Further, the role of myocyte health in axonal survival and the putative retrograde cellular pathophysiology remains unresolved. Future studies will be required to determine the precise mechanisms by which proteins that regulate motoneuron and skeletal muscle interaction are affected by unfolded proteins and ER stress leading to muscle atrophy and weakness.

## Conclusion

In summary, our findings show that ER stress is present in skeletal muscle of transgenic ALS mice starting at an early, pre-symptomatic age. The ER stress sensors (PERK, IRE1α), ER chaperones (Grp78/BiP, PDI), and ER stress-induced apoptotic mediator (CHOP) are activated and associated with disease pathophysiology and inhibition of protein translation in skeletal muscle. Additionally, we show that ER stress activation is greatest in glycolytic skeletal muscle and muscles of highest contractile activity demand. These data suggest that ER stress induces an early cellular pathology in skeletal muscle that may contribute to the atrophy in ALS.

## Authors Contribution

ERC designed the study, established the G93A*SOD1 animal colony and completed animal dissections. DC assisted in maintaining colonies and completed muscle tissue analyses. YW advised on muscle sample preparation for mass spectrometry, completed mass spectrometry analysis and interpretation of MS data. DC, YW and ERC made figures, analyzed the data, and interpreted the findings. DC and ERC wrote the manuscript. DC, YW and ERC edited the manuscript. All authors read and approved the final manuscript.

## Conflict of Interest Statement

Eva R. Chin and Dapeng Chen are Inventors on a pending patent which includes some of these data. Eva R. Chin is the Founder and Chief Scientific Officer of MyoTherapeutics, a University of Maryland-based start-up company. Yan Wang declares that the research was conducted in the absence of any commercial or financial relationships that could be construed as a potential conflict of interest.

## Abbreviations

ALS: amyotrophic lateral sclerosis
SOD1: Cu/Zn superoxide dismutase
ER: endoplasmic reticulum
UPR: unfolded protein response
PERK: protein kinase RNA-activated (PKR)-like ER kinase
IRE1α: inositol-requiring kinase 1-alpha
eIF2α: eukaryotic initiation factor 2 alpha
PDI: protein disulfide isomerase
Grp78/BiP: 78 kDa glucose-regulated protein and immunoglobulin binding protein
CHOP: C/EBP-homologous protein
SDS-PAGE: dodecyl sulfate polyacrylamide gel electrophoresis
WG: white gastrocnemius
RG: red gastrocnemius
DIA: diaphragm
HRT: heart
LIV: liver.
