# Supplementary material for: Activation of the endoplasmic reticulum stress response in skeletal muscle of G93A*SOD1 amyotrophic lateral sclerosis mice
Source: Front Cell Neurosci. 2015 May 18;9:170. doi: 10.3389/fncel.2015.00170 (PMC4435075; doi:10.3389/fncel.2015.00170)
Supplement: Supplementary file 1 [file Table_1.PDF]

Table 1 2-way ANOVA results for proteins analyzed

	GENOTYPE Main Effect	AGE Main Effect	Genotype * Age Interaction Effect	Genotype * Age Tukey Post-hoc (Age)			Genotype		
				**ALS** *70d vs 90d*	**ALS** *70d vs. 120-140d*	**ALS** *90d vs. 120-140d*	**70 d WT vs. ALS**	**90 d WT vs. ALS**	**120-140 d WT vs. ALS**
PERK	P=0.001<br><br>WT vs. ALS p p=0.001	P=0.463	p=0.332	P=0.068	P=0.079	P=0.891	P = 0.015	P = 0.025	P = 0.001
p-PERK/total PERK	P=0.044<br><br>WT vs. ALS p p=0.044	P=0.049<br><br>70d vs. 90d p=0.083<br>70d vs. 120-140d p=0.016<br>90d vs. 120-140d p <0.304	P=0.100	P=0.049	P=0.002	P=0.062	P = 0.60	P = 0.45	P = 0.012
p-eIF2α	P=0.009<br><br>WT vs. ALS p p<0.009	P=0.244	P=0.204	P=0.227	P=0.018	P=0.101	P = 0.005	P = 0.048	P = 0.011
IRE1α	p = 0.000..<br><br>WT vs. ALS p<0.001	P = 0.244	P = 0.088	P = 0.087	P = 0.008	P = 0.119	P = 0.043	P = 0.0005	P = 0.0002
XBP-1s	P = 0.000…<br><br>WT vs. ALS p<0.001	P = 0.028<br><br>70d vs. 90d p=0.012<br>70d vs. 120-140d p=0.024<br>90d vs. 120-140d p = 0.668	P = 0.016	p = 0.002	P = 0.002	P = 0.932	P= 0.967	P = 0.001	P = 0.000…
PDI	p<0.001	p<0.001	P=0.001	P=0.004	P=0.001	P<0.001	P = 0.11	P = 0.93	P = 0.001

	WT vs. ALS p<br>p<0.001	70d vs. 90d<br>p=0.035<br>70d vs. 120-140d<br>p=0.014<br>90d vs. 120-140d<br>p <0.001							
Grp78/BiP	p<0.001<br><br>WT vs. ALS p<br>p<0.001	p<0.001<br><br>70d vs. 90d<br>p=0.803<br>70d vs. 120-140d<br>p=0.001<br>90d vs. 120-140d<br>p <0.001	p<0.001	P=0.803	p <0.001	p <0.001	P = 0.00565	P = 0.025	P = 0.0053
CHOP - WG	P=0.001<br><br>WT vs. ALS p<br>p=0.001	P=0.001<br><br>70d vs. 90d<br>p=0.791<br>70d vs. 120-140d<br>p=0.001<br>90d vs. 120-140d<br>p =0.001	P=0.001	P=0.772	P<0.001	P<0.001	P = 0.041	P = 0.034	P = 0.019
CHOP-DIA	P=0.014<br><br>WT vs. ALS p<br>p=0.014	P=0.063<br><br>70d vs. 90d<br>p=0.754<br>70d vs. 120-140d<br>p=0.038<br>90d vs. 120-140d<br>p =0.043	P=0.063	P=0.0620	P=0.004	P=0.005	P = 0.008	P = 0.001	P = 0.10
